# Genome-wide data reveal bi-direction and asymmetrical hybridization origin of a fern species *Microlepia matthewii*

**DOI:** 10.3389/fpls.2024.1392990

**Published:** 2024-07-08

**Authors:** Jun-Jie Luo, Hui Shang, Zhi-Qing Xue, Ying Wang, Xi-Ling Dai, Hui Shen, Yue-Hong Yan

**Affiliations:** ^1^ Eastern China Conservation Centre for Wild Endangered Plant Resources, Shanghai Chenshan Botanical Garden, Shanghai, China; ^2^ College of Life Sciences, Shanghai Normal University, Shanghai, China; ^3^ Middle School Department, Songjiang Experimental School Affiliated To Shanghai University of International Business and Economics (SUIBE), Shanghai, China; ^4^ Shanghai Chenshan Science Research Center, CAS Center for Excellence in Molecular Plant Sciences, Chinese Academy of Sciences, Shanghai, China; ^5^ Key Laboratory of National Forestry and Grassland Administration for Orchid Conservation and Utilization, The National Orchid Conservation& Research Center of Shenzhen, Shenzhen, Guangdong, China

**Keywords:** dd-GBS, dennstaedtiaceae, gene flow, pteridophyte, SNPs, species diversity

## Abstract

**Introduction:**

Natural hybridization is common and plays a crucial role in driving biodiversity in nature. Despite its significance, the understanding of hybridization in ferns remains inadequate. Therefore, it is imperative to study fern hybridization to gain a more comprehensive understanding of fern biodiversity. Our study delves into the role of hybridization in shaping fern species, employing *Microlepia matthewii* as a case study to investigate its origins of hybridization.

**Methods:**

We performed double digest Genotyping-by-sequencing (dd-GBS) on M. matthewii and its potential parent species, identifying nuclear and chloroplast SNPs. Initially, nuclear SNPs were employed to construct the three cluster analysis: phylogenetic tree, principal component analysis, and population structure analysis. Subsequently, to confirm whether the observed genetic mixture pattern resulted from hybridization, we utilized two methods: ABBA-BABA statistical values in the D-suite program and gene frequency covariance in the Treemix software to detect gene flow. Finally, we employed chloroplast SNPs to construct a phylogenetic tree, tracing the maternal origin.

**Results and discussion:**

The analysis of the nuclear SNP cluster revealed that M. matthewii possesses a genetic composition that is a combination of *M. hancei* and *M. calvescens*. Furthermore, the analysis provided strong evidence of significant gene flow signatures from the parental species to the hybrid, as indicated by the two gene flow analyses. The samples of *M. matthewii* cluster separately with *M. hancei* or *M. calvescens* on the chloroplast systematic tree. However, the parentage ratio significantly differs from 1:1, suggesting that *M. matthewii* is a bidirectional and asymmetrical hybrid offspring of *M. hancei* and *M. calvescens*.

## 1 Introduction

A significant proportion of identified species have originated from hybridization, with at least 25% of plant species and 10% of animal species, predominantly the youngest ones, being involved in hybridization and potential introgression with other species (Mallet, 2005). Natural hybridization denotes the gene flow between populations that have naturally undergone genetic differentiation under natural conditions (Arnold, 1992 and 1997). It constitutes a pivotal factor in the speciation and evolution of vascular plants (Abbott et al., 2016), particularly in ferns (Barrington et al., 1989; Sigel, 2016), and greatly influence on species diversity. On one hand, hybridization has the potential to lead to the reverse speciation of taxa in the process of species differentiation, giving rise to the formation of extensive hybrid clusters that may contribute to a reduction in species diversity (Seehausen, 2006; Grant and Grant, 2014). Moreover, it has the potential to contribute to the extinction of specific rare species or populations (Balao et al., 2015). On the other hand, natural hybridization is a vital process of speciation to increase species diversity. One mechanism is allopolyploid introgression (Soltis et al., 2014), exemplified by the tetraploid *Primula kewensis* resulting from the hybridization and genome duplication of two diploid species, *P. verticillata* and *P. floribunda* (Ramsey and Schemske, 2002). Another mechanism is autopolyploid hybridization adaptation introgression. For instance, the sunflower (*Helianthus annuus*) thrives in clayey soils, while its close relative, the prairie sunflower (*H. petiolaris*), flourishes in sandy soils. Consequently, three natural hybridizations (*H. anomalus*, *H. deserticola*, and *H. paradoxus*) have emerged, each uniquely adapted to a specific environment (Rieseberg, 1991). Therefore, recognizing and identifying these types of hybridization is essential for understanding the origin of species diversity.

Ferns are highly susceptible to natural hybridization due to ineffective reproductive isolation mechanisms (Knobloch, 1976; Barrington et al., 1989). This is evident even between distantly related taxa, exemplified by the natural hybrid offspring *Cystocarpium roskamianum*. Its parent specie belong to different generas, *Cymnocarpium* and *Cystopteris*, and had diverged from each other approximately 60 million years ago (Rothfels et al., 2015). This results in a high proportion of hybridized fern species. A survey in Japan identified 371 interspecific hybrids in addition to the 721 native, non-hybrid taxa comprising the fern and lycophyte flora of Japan (Ebihara and Nitta, 2019). In Hawaii, the number of hybridized species is 37 out of a total of 221 species of fern and fern allies (Palmer, 2003). The identification of fern hybrids is crucial for recognizing fern diversity. However, research on fern hybrids in some areas has been insufficiently executed. For instance, according to the *Flora of China* (Wu et al., 2013), only 62 fern hybrids have been identified and hypothesized, constituting less than 3% of the total number of 2254. This figure is lower than the preliminary estimate of about 500 naturally hybridized ferns in China by Yan et al. (2016), which was based on conditions in neighboring regions such as Japan and field observations. In recent years, gene fragments from the chloroplast genome, which has been proven to exhibit maternal inheritance in ferns (Gastony and Yatskievych, 1992; Vogel et al., 1998; Guillon and Raquin, 2000), and nuclear genes (exhibiting biparental inheritance) have been utilized to identify the hybrid origin and parentage of many fern species. Examples include *Adiantum ×meishanianum* (Zhang et al., 2014; Shang et al., 2016), *A. ×ailaoshanense* (Wang et al., 2015a, b), and *Microsorum ×tohieaense* (Nitta et al., 2018). In the majority of these studies, hybridization occurred in a unidirectional manner, where either the mother or father was clearly identified as the definite parent, rather than in the opposite direction. Additionally, using molecular markers of biparental inheritance (chloroplast gene *rbcL* and the nuclear gene *pigC*), along with the C-value of DNA reflecting cellular ploidy, Hori et al. (2014) revealed reticulate evolutionary relationships among 11 species, including the *Dryopteris varia* complex and its related species, suggesting that hybridization in ferns is common. Furthermore, Yi et al. (2023) employed restriction-site-associated DNA sequencing (RAD-seq) and integrated phylogenomics and population genomic analyses to investigate the phylogenetic relationships and evolutionary history of 16 scaly tree ferns (Cyatheaceae) from China and Vietnam, which revealed genome-wide evidence for prevalent hybridization not only between closely related species but also between distantly related species from different genera.

**Figure 1:**
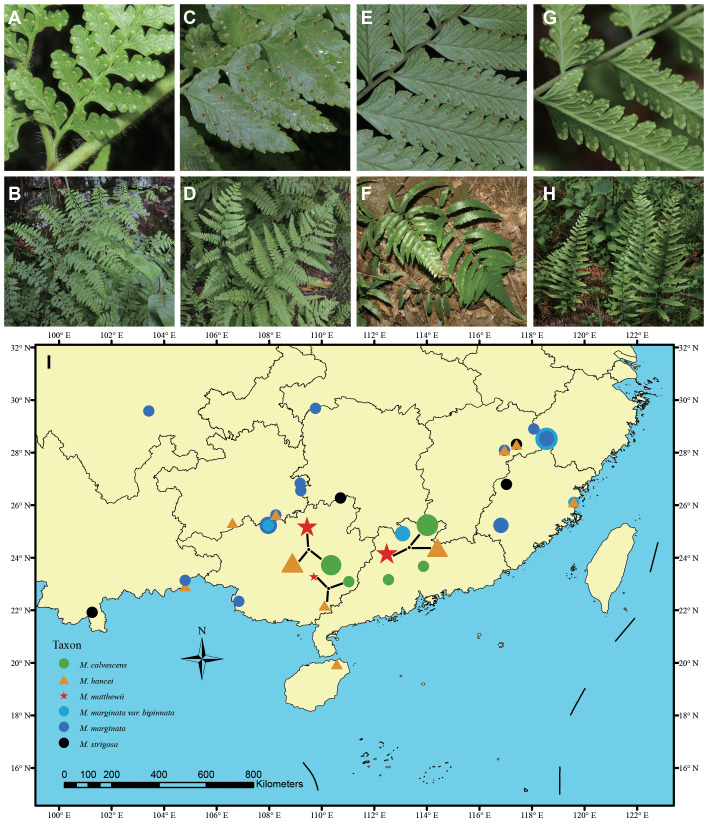
Photos of living plants of hybrid and potential parent species in this study. **(A, B)** *Microlepia hancei*. **(C, D)** *M. matthewii*. **(E, F)** *M. calvescens*. **(G, H)** *M. marginata.* Photo credits by Hong-Jin Wei. **(I)** The sampling map uses different symbols to represent various species, with the size of each symbol indicating the sample size of each population.

*Microlepia* C. Presl is a member of the Dennstaedtiaceae family, which constitutes one of the six main clades (Suborders) within the Polypodiales order (PPG I, 2016; Du et al., 2021). Dennstaedtiaceae comprises eleven small genera, with only three, namely *Microlepia*, *Hypolepis*, and *Dennstaedtia*, having more than 50 species each (Schwartsburd et al., 2020; Triana-Moreno et al., 2023). *Microlepia* encompasses a wide range of species with significant morphological diversity, including the 1–4-pinnately compound lamina (Moore, 2010; Yan et al., 2013; Luo, 2018), six different leaf epidermal and stomatal characteristics (van Cotthem, 1970), significant variation in chromosome number (Nakato and Serizawa, 1981; Nakato and Ebihara, 2011), and diverse spore ornamentation, which can be inner lophate or reticulate and outer sericate or capillate (Luo et al., 2018). Hybridization may contribute to the observed high diversity in *Microlepia*. Several studies (Shang et al., 2015 and 2016; Schwartsburd et al., 2020) have indicated the presence of numerous hybrids based on morphological and chloroplast fragment analyses, and these hybrids may share a maternal plastid genome with morphologically distinguishable species. In the case observed in *Microlepia*, *M. matthewii* is found on branches of various other species, while *M. krameri* and *M. herbacea* are in similar situations (Wang, 2016; Luo et al., 2018). In this study, we selected *M. matthewii* as a representative species to investigate the role of hybridization in generating diversity within the *Microlepia* genus. Morphologically, *M. matthewii* displays intermediate traits between *M. hancei* and *M. marginata* (**Figures 1A-H**). Additionally, spores of *M. matthewii* are observed to be deformed and incapable of germination (Luo, 2018). Considering its abnormal chloroplast phylogenetic position, where several samples of *M. matthewii* clustered with *M. hancei* while a small number clustered with *M. marginata*, we hypothesize that *M. matthewii* may be a hybrid with either *M. hancei* or *M. marginata* as the maternal species.

However, the chloroplast genome has only a small number of variant sites, exhibits maternal inheritance, and is relatively conserved compared to the entire genome. While some studies have considered nuclear genes (Zhang et al., 2014; Wang et al., 2015a; Shang et al., 2016; Nitta et al., 2018), relying solely on a few gene fragments presents several limitations. These include the inability to detect hybridization beyond F1 generations or minimal introgressive hybridization. Additionally, the selected gene segments often exhibit limited variation, making it challenging to accurately infer the origin of hybrids. Therefore, genetic markers at the whole-genome level are essential. More recently, the use of high-throughput sequencing, including reduced representation genome sequencing (also known as simplified genome sequencing) and genome re-sequencing technologies, has facilitated the detection of hybridization or gene flow. Furthermore, in this study, *M. marginata*, as the potential parent of *M. matthewii*, is a complex comprising numerous variations (Yan et al., 2013; Wang and Liu, 2023). However, it cannot be distinguished using several gene fragments alone (Luo et al., 2018). The genomic evidence should be helpful to determine the relationships among the species within this complex, which are not well resolved in the phylogenetic tree based on chloroplast fragments. Overall, in this study, employing a type of simplified genome sequencing—double digest Genotyping-by-sequencing (dd-GBS), we aims to address the scientific questions: 1) is *M. matthewii* of hybrid origin? And 2) what are the respective paternal and maternal species of *M. matthewii*?

## 2 Material and methods

### 2.1 Material

In this study, we sampled a total 88 material, including 21 individuals of suspected hybrid *M. matthewii* and its potential parents (19 individuals of *M. hancei* and 44 individuals of *M. marginata*) based on the hypotheses outlined in the introduction section of this study and findings from previous research (Wang, 2016; Luo et al., 2018). *M. marginata* includes the original variant, var. *calvescens* (sometimes treated as *M. calvescens* in some literature, e.g. Liu et al., 2016; Wei and Zhang, 2016; Fraser-Jenkins et al., 2017), var. *bipinnata*, with an additional four individuals of *M. strigosa* as outgroups based on previous study (Luo et al., 2018). Most samples (60 individuals, 68.2% of the total) were collected at the population level, while the remaining samples were obtained from previous field collections stored in the National Wild Plant Germplasm Resource Center for Shanghai Chenshan Botanical Garden (ZWGX2202). The sampling locations are represented on the map (**Figure 1I**), and further details regarding the sampling are listed in **Supplementary Table S1**.

These specimens were field-collected and dried using silica gel. Voucher specimens were curated at Chenshan Herbarium (CSH). DNA extraction from silica gel-dried leaves followed the modified CTAB method, with subsequent determination of DNA concentration and total amount. Samples passing the test proceeded to double digest Genotyping-by-sequencing (dd-GBS) library construction, involving double-enzymatic (Msel-Taqal) digestion and adapter attachment to the cleavage site ends of digested fragments. Primers designed based on adapter sequences were used for fragment amplification. After passing quality control, bipartite sequencing was conducted on the Illumina HiSeq X Ten platform (Illumina, USA), capturing approximately 150 bases of sequence on either end. Raw data underwent adapter trimming and quality screening to obtain clean data. Reads of low quality, including those with more than 40% of nucleotides having a quality value lower than 15, more than 10% of N nucleotides, or a length less than 30bp, were discarded.

### 2.2 SNP calling

The chloroplast and nuclear SNP datasets were obtained separately. To call chloroplast SNPs, we used the published plastid genome of *M. marginata* (GenBank assembly accession: MT130649; Du et al., 2021) as the reference. The clean data of 88 samples were then mapped to this reference using BWA v.0.7.12-r1039 (Li and Durbin, 2009) with maximal exact matches. The resulting BAM files were directly used in the standard GATK Best Practices workflow to call variants via the GATK HaplotypeCaller algorithm. Default GATK program settings were applied to filter the variants, and InDel variants were excluded from our study. In the end, we obtained 4649 chloroplast SNPs.

Nuclear SNPs were called by assembling the retained clean reads using the *de novo* assembly pipeline Stacks v2.1 (Rochette et al., 2019), and before calling, reads that could be mapped to the chloroplast reference were excluded. We required at least six identical reads (option –m 6) to create a stack for each individual using ustacks. A catalog of all loci across populations was then constructed using cstacks. After matching each sample against the catalog with sstacks, tsv2bam and gstacks were executed to incorporate paired-end reads, identify, and phase the SNPs. The variant call format (VCF) of called variants was exported using populations. The final dataset for subsequent analysis was filtered by vcftools with a minor allele frequency of 0.01, a maximum missing rate of 0.5, and a minimum depth of five. In the end, we obtained 1274 nuclear SNPs.

### 2.3 Nuclear SNP cluster using IQtree, admixture and principal component analysis

Phylogenetic trees were constructed from a nuclear SNP dataset using maximum likelihood (ML) concatenation analysis. The best-fit nucleotide substitution models were selected based on the Bayesian information criterion (BIC) using ModelFinder (Kalyaanamoorthy et al., 2017) implemented in IQTREE v2 (Nguyen et al., 2015). IQTREE was executed with the ascertainment bias correction (+ASC) model (Lewis, 2001) for the nuclear SNP dataset, and 1000 ultrafast bootstrap replicates were performed.

Population structure analysis was performed using ADMIXTURE v1.3.0 (Alexander et al., 2009). The number of pre-defined genetic clusters ranged from K = 1 to K = 7, determined based on the number of species and presumed genetic components, and adjusted according to the final results. The best-fitting model was determined using cross-validation (CV) error.

Plink v2.0 (Purcell et al., 2007) was applied to conduct principal component analysis (PCA) on the nuclear SNPs. Each sample was plotted based on the first two principal components (PCs), and the resulting figure was generated using R 4.1.3 with the ggplot2 package (https://ggplot2.tidyverse.org).

### 2.4 Gene flow test using treemix and Dsuite

To assess historical introgression between species in the nuclear genome, we employed Patterson’s D-statistic (ABBA-BABA statistics; Durand et al., 2011) in the Dsuite program (Malinsky et al., 2021) and gene frequency covariance in the treemix software (Pickrell and Pritchard, 2012).

For Treemix analysis, the vcf file was obtained using vcftools and converted to the Treemix format using a Python script (https://github.com/wk8910/bio_tools/tree/master/03.treemix, accessed on 7 May 2023). Then, the analysis was conducted incrementally, considering pre-defined numbers of migration events (1-5) using the software Treemix. The increase in explained variation was compared for each number of migration events to infer the best result. The tree for the best result was visualized in R 4.1.3.

In Dsuite analysis, we assessed admixture across the *M. hancei* to *M. marginata* complex utilizing Patterson’s D (Green et al., 2010; Durand et al., 2011) and the f-branch (fb) statistic (Malinsky et al., 2018) with Dsuite v0.4r38 (Malinsky et al., 2021). To determine Dmin, representing the minimum allele sharing for all trios of ingroup lineages (n = 5), we employed Dtrios from Dsuite with the SNP dataset and the treemix phylogeny, irrespective of any assumptions about the tree topology. The fb statistic with Fbranch from Dsuite summarized rates of introgression. The generated “.tree” file by Dtrios and the treemix phylogeny were employed, and resulting fb statistics were plotted on the treemix phylogeny using “dtools.py”. *M. strigosa* served as the out-group.

### 2.5 Chloroplast phylogeny to identify maternal parents

Phylogenetic analyses of the chloroplast SNP dataset were conducted employing three different methods: maximum parsimony (MP), maximum likelihood (ML), and Bayesian Inference (BI). These analyses were performed using PAUP * 4.0b10 (Swofford, 2003), RAxML-HPC (Stamatakis, 2006), and MrBayes v3.2.5 (Huelsenbeck and Ronquist, 2001; Ronquist et al., 2012), respectively.

The ML analyses were performed using RAxML-HPC (Stamatakis, 2006) with ML tree searches and bootstrapping. The default model of -m GTRCAT was applied, along with 1000 rapid bootstrap analyses. A one-time search for the best-scoring tree was conducted (Stamatakis et al., 2006).

The MP analyses were carried out in PAUP* ver. 4.0b10 (Swofford, 2003) with 1000 tree-bisection-reconnection (TBR) searches. One thousand replicates were performed, each with 10 TBR searches, and a maximum of 100 trees were held per TBR search.

The best-fitting likelihood model for Bayesian analyses was chosen based on the Bayesian information criterion using jModeltest2 (Darriba et al., 2012). Bayesian inference (BI) was performed using MrBayes 3.2.5 (Huelsenbeck and Ronquist, 2001; Ronquist et al., 2012). We executed two independent runs, each employing four chains – one cold and three heated. The temperature parameter was set at 0.2, and the transition/transversion rate ratio was designated as beta, while the priors remained at their default settings. At the outset of each run, a random tree was initialized, and subsequently, every 1000 generations, a single tree was sampled, totaling 10,000,000 generations. The evaluation of convergence and stationarity was conducted using Tracer version 1.4 (Rambaut and Drummond, 2007), and to ensure the convergence of runs, the initial 25% of trees were discarded as burn-in. Subsequently, the remaining trees were employed to compute posterior probabilities (PP) for the majority-rule consensus topology.

The G-test was used to test the parentage ratio of the 21 individuals of the suspected hybrid *M. matthewii* (whether the ratio of *M. calvescens: hancei* significantly deviation from the expected 1:1 ratio). This analysis was conducted using the RVAideMemoire package (Herve, 2023) in R 4.1.3.

Furthermore, Bayesian Species Delimitation analyses (BFD) were conducted using the chloroplast SNP dataset to assess whether *M. calvescens* is a distinct species compared to other *M. marginata* variants, without considering hybridization. The nexus file was used to create the BFD input xml files in BEAUti v.2.4.5 (Bouckaert et al., 2014). We tested five different species assignment models, either combining the variants of *M. marginata* differently or treating them as distinct units. The BFD analyses were executed in SNAPP v.1.2.5 (Bryant et al., 2012), utilizing 12 initialization steps and chain-lengths of one million for each clade model. The remaining settings followed the guidelines set forth by Brandrud et al. (2020).

## 3 Result

### 3.1 SNP cluster: phylogenetic tree, admixture, and PCA

**Figure 2:**
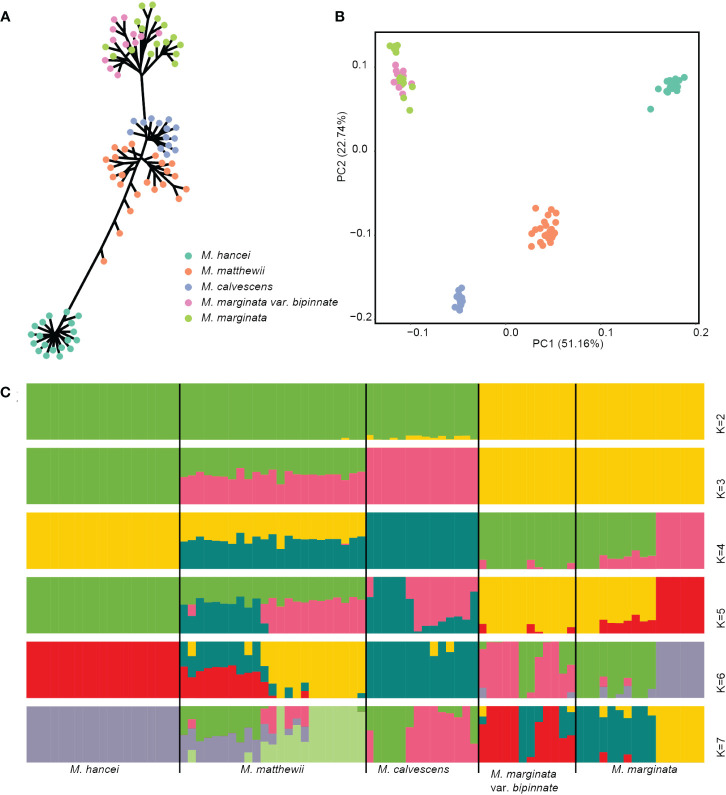
Genetic cluster based nuclear SNPs of four *Microlepia* species in this study. **(A)** Phylogenetic tree (unrooted). **(B)** PCA plot. Different colors representing different species in **(A, B)**. **(C)** Genetic structure output from admixture. The length of each colored segment represents the proportion of the individual’s genome from K = 2 to 7 ancestral genetic groups. The samples are grouped by species and are depicted with different colors representing different genetic components.

On the nuclear SNP phylogenetic tree (**Figure 2A**), the samples of *M. hancei*, *M. marginata* (excluding *M. calvescens*) and *M. calvescens* formed monophyletic groups separately (therefore, in the following text, any reference to *M. marginata* will exclude *M. calvescens*). However, *M. matthewii* did not form a monophyletic clade.

The PCA analysis demonstrated a clear separation of the 84 individuals into four lineages on the first two axes (PCs), which accounted for 73.9% of the total variation (see **Supplementary Figure S1A**). These lineages corresponded to *M. hancei*, *M. marginata*, *M. calvescens*, and *M. matthewii* (positioned between *M. calvescens* and *M. hancei*) (**Figure 2B**).

The admixture analysis confirmed the patterns observed in PCA (**Figure 2C**), especially at K = 3, effectively distinguishing *M. hancei*, *M. marginata*, *M. calvescens*, and *M. matthewii* as a mixture of *M. hancei* and *M. calvescens*. The result at K = 4 showed a division of *M. marginata* into two genetic components. However, based on the CV error, K = 5 was determined as the optimal value (see **Supplementary Figure S1B**), leading to the further division of *M. calvescens* into two genetic components. Conversely, all *M. marginata* variations except *M. calvescens* grouped together in the K = 2 and K = 3 results.

### 3.2 Gene flow: treemix, Dsuite

**Figure 3:**
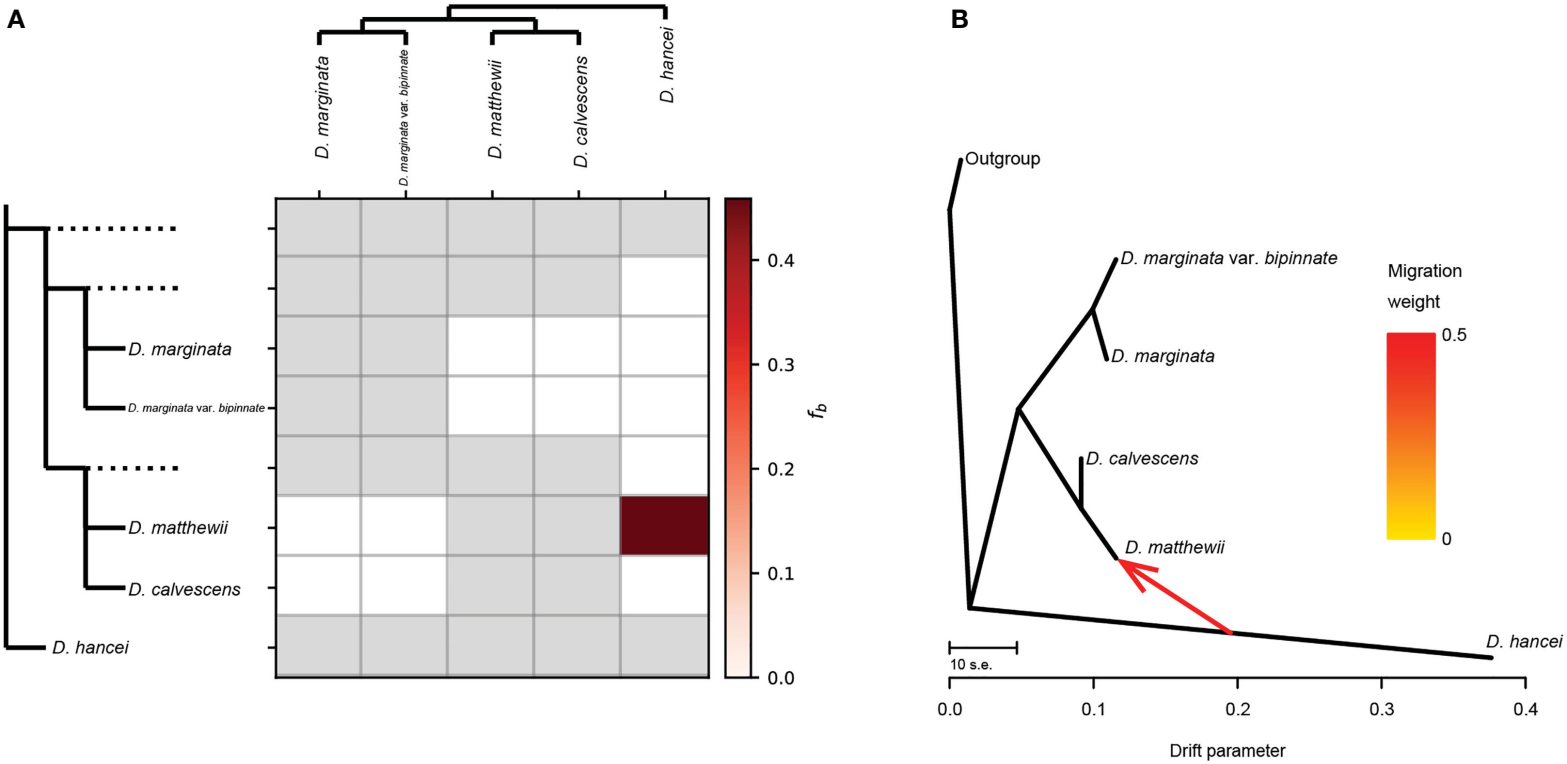
Gene flow between *Microlepia* species in this study. **(A)** Gene flow signals detected by Dsuite. The heatmap shows the magnitude of the fb ratio between each branch (left) and the sample (top). Grey squares indicate comparisons that cannot be made. **(B)** Gene flow depicted as the maximum-likelihood tree produced in Treemix.

The ABBA-BABA tests revealed that three out of the ten tested four-taxon phylogenies, considering triplets involving *M. matthewii* and *M. hancei*, showed a significant signal of introgression (*P* < 0.05, non-zero D-statistics) (**Table 1**). D-statistics ranged from 0.88 to 0.92 for all the significant tests. Additionally, one gene flow from *M. hancei* to *M. matthewii* was identified on the f-branch (fb) statistic plot (**Figure 3A**).

**Table 1:** D-statistics and F4-ratios from Dsuite for all species trios in this study.

P1	P2	P3	Dstatistic	Z-score	p-value	f4-ratio	BBAA	ABBA	BABA
calv	bipi	hanc	0.198543	0.9685	0.332795	0.0155909	15.0716	5.28774	3.53588
marg	bipi	calv	0.0561275	0.509763	0.610218	0.0247423	81.6818	15.6793	14.0127
matt	calv	bipi	0.0749805	1.19853	0.230711	0.0465152	85.2588	30.856	26.5515
marg	bipi	hanc	0.272945	1.22534	0.220446	0.00816316	30.9372	2.29281	1.30956
**bipi**	**matt**	**hanc**	**0.887466**	**29.1721**	**2.30E-16**	**0.480265**	**8.77293**	**61.0058**	**3.63726**
marg	bipi	matt	0.0623555	0.605526	0.54483	0.018913	88.6595	15.3525	13.5503
calv	marg	hanc	0.109078	0.590595	0.554792	0.00988587	15.2011	5.67694	4.56029
**calv**	**matt**	**hanc**	**0.919123**	**35.1084**	**2.30E-16**	**0.458846**	**15.9857**	**58.1671**	**2.45133**
matt	calv	marg	0.0753037	1.07185	0.283786	0.0482839	88.5927	30.8781	26.5533
**marg**	**matt**	**hanc**	**0.882272**	**30.5306**	**2.30E-16**	**0.482682**	**8.62111**	**62.1501**	**3.8872**

calv, *M. calvescens*; bipi, *M. marginata* var *bipinnate*; hanc, *M. hancei*; marg, *M. marginata*; matt, *M. matthewii*. Bold text indicate the significant models (p<0.05).

The general topology of the Treemix ML tree (**Figure 3B**) was consistent with the phylogenetic relationship recovered from the other phylogenetic analysis. One migration event between the *M. matthewii* and *M. hancei* improved with the explained variation from the initial 96% (with zero migration events allowed) to 99.96% (with one migration event allowed). The variation of further migration events (two to four) are 99.98%, 99.99% and 100%. As a max explained variation improving, the scenario with only one migration event was chosen to be the best model.

### 3.3 Chloroplast phylogeny

**Figure 4:**
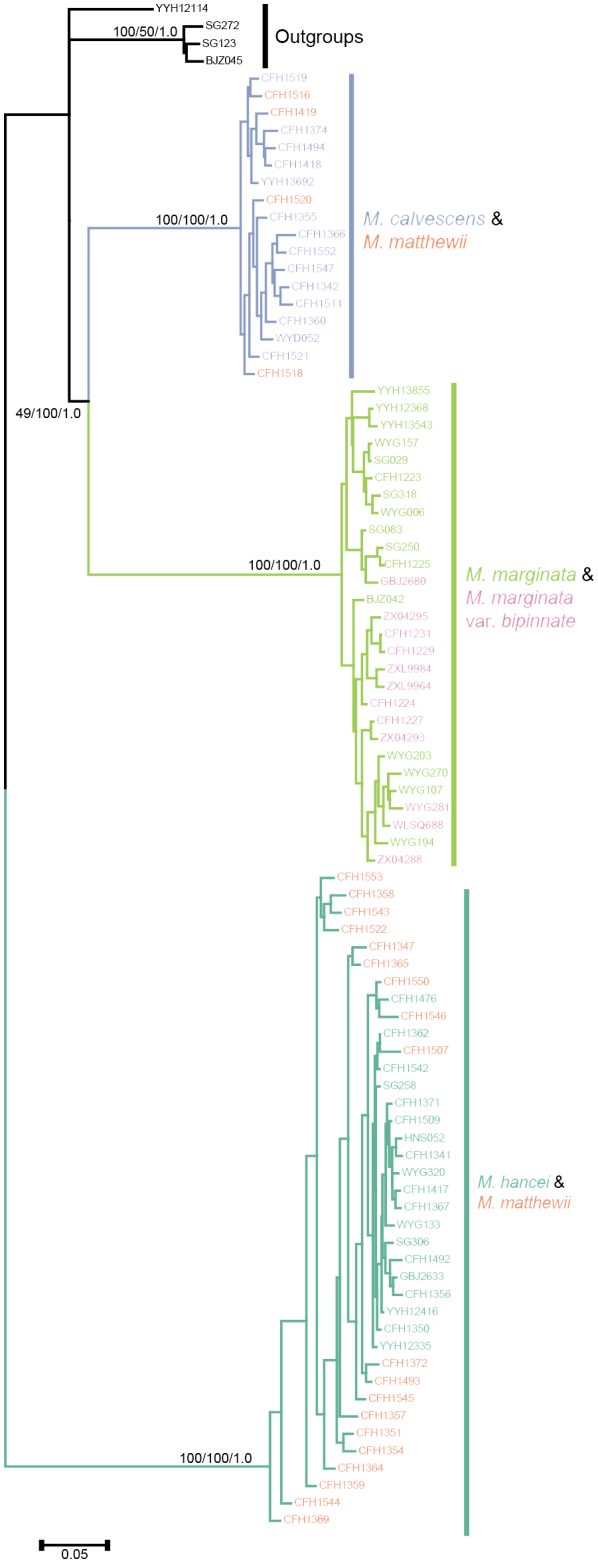
Phylogenetic tree based chloroplast SNPs. Different colors representing different species same as **Figures 2A, B**.

By employing the chloroplast genome of *M. marginata* as a reference, a comprehensive set of 4,649 SNP loci was acquired. Subsequent tree construction using various methodologies, including maximum parsimony, maximum likelihood, and Bayesian methods, consistently revealed similar topologies (**Figure 4**). Excluding outgroups, three well-supported monophyletic lineages were identified within the inner group. *M. calvescens* constituted a distinct monophyletic group, while the other variants of *M. marginata* formed a sister group without *M. calvescens*, representing a separate monophyletic lineage. The third monophyletic lineage comprised *M. hancei*.

The two most optimal models identified in the Bayesian Species Delimitation (BFD) analyses both supported the topology in which *M. calvescens* is considered a distinct species, separate from other *M. marginata* variants, as indicated by the maximum marginal likelihood estimates (MLE) shown in **Table 2**. The MLEs of the first and second optimal models were very close (Δ<10), and significantly higher (Δ>1000) than that of the third-ranked model.

**Table 2:** Results of Bayesian Species Delimitation analyses (BFD) in this study.

No.	Models	MLE
1	hanc, (stri, (marg+calv+bipi))	-30257.5917
2	hanc, (stri, (calv, (marg +bipi)))	-28023.9195
3	hanc, (stri, (calv, (marg, bipi)))	-28016.4879
4	hanc, (stri, (bipi, (calv+ marg)))	-30046.2461
5	hanc, (stri, (marg, (calv+bipi)))	-29894.9487

MLE, marginal likelihood estimate; calv, *M. calvescens*; bipi, *M. marginata* var *bipinnate*; hanc, *M. hancei*; marg, *M. marginata*; stri, *M. strigosa*. In different models, hypotheses connected by a “+” are considered as conspecifics, while those separated by a “,” are considered as different taxa.

Within these lineages, four individuals in *M. matthewii* clustered in the clade of *M. calvescens*, while the remaining 17 individuals clustered in the *M. hancei* clade, resulting in a significant deviation from the expected 1:1 ratio (G test: G= 8.6618, *P*= 0.003249).

## 4 Discussion

### 4.1 Genetic differentiation and mixture

The three cluster analyses conducted in this study—phylogeny, structural analyses, and PCA—clearly depict a mixture pattern for *M. matthewii*, suggesting the possibility of it being a hybrid species. Further details are discussed below:

The result of admixture analyses for K = 2 didn’t agree with the phylogenetic tree, as the *M. marginata* complex separated first, and *M. hancei*, *M. calvescens* and *M. matthewii* clustered together in another group. In many studies, the first run of structure at K = 2 tends to be the earliest of the two major branches to be separated or not genetically mixed (e.g., Yi et al., 2023) and often exists as the optimal K (e.g., Chattopadhyay et al., 2019; Xue et al., 2021; Encinas-Viso et al., 2022) because there are generally much longer branch lengths between the two main clades than within them. However, in this study, *M. calvescens* did not cluster with its closest relative on the phylogenetic tree, *M. marginata*, but instead with *M. hancei* at K = 2. This may be attributed to strong gene flow between *M. calvescens* and *M. hancei*, causing the two, along with their hybrid offspring, to cluster into a single genetic component first. Additionally, the CV error of K = 2 is very high and not optimal, predicting the implausibility of the structure.

The outcome from K = 3 indicates that *M. calvescens* forms a unique evolutionary cluster on the structure diagram, suggesting it should be classified as a distinct species. The remaining variants of *M. marginata*, including the original variant, var. *bipinnata*, and so forth, appear identical. The phylogenetic tree verifies the separation of *M. calvescens* and the other *M. marginata* varieties as two independent sister groups, both forming monophyletic clusters. Additionally, the principal component analysis (PCA) results indicate that *M. calvescens* and other *M. marginata* variants constitute two distinct groups.

The results of K = 4 for *M. marginata* reveal two distinct genetic groups, implying further differentiation within the species *M. marginata* or among these variants. However, the genetic groups do not correspond to the variants, suggesting that the differentiation is incomplete or ongoing and there is no evidence of new species arising at the present stage. And the results of PCA also did not support differentiation, with all individuals clustered together closely. Although some individuals in *M. marginata* are slightly separated on the PCA plot, consistent with the results of K = 4, they are so close that *M. marginata* is still considered a clustered entity.

The results of K = 5 for *M. calvescens* also reveal two distinct genetic groups, but this is not supported by the PCA, where all the plots clustered together.

Although the optimal K value is five with the lowest CV error value, the CV error values for K values ranging from three to six are very close to each other (**Supplementary Figure S1**, 0.28398, 0.27836, 0.26708, and 0.27742). Based on the results of the PCA and phylogenetic tree, K = 3 appears to be the most appropriate choice. Most importantly, the findings from K = 3 to K = 5 analyses reveal two significant points: 1) *M. calvescens* constitutes a distinct genetic component separate from other *M. marginata* variants, consistent with both the phylogeny and BFD analyses; 2) The presence of a mixture of *M. calvescens* and *M. hancei* genetic components in *M. matthewii* suggests a potential hybridization event between these two species. This speculation is also supported by the fact that *M. matthewii* lies between *M. calvescens* and *M. hancei* on the PCA plot.

### 4.2 Gene flow

Hybridization, involving gene flow between species, can lead to a mixed genetic structure. However, it is not the only cause. Two other mechanisms, namely ghost admixture and recent bottleneck effects, can also result in a mixed pattern (Lawson et al., 2018). Therefore, classical methods such as phylogeny, structural analyses, and PCA are currently insufficient for fully identifying instances of species hybridization. Gene flow assays are necessary tools that must be employed to augment these methods.

Therefore, we employed two methods to calculate gene flow to test whether the mixed pattern of *M. matthewii* is caused by hybridization. One of them, treemix, is based on gene frequency covariance. The presence of gene flow is indicated if the actual value is less than the estimated value, as gene flow can reduce gene frequency covariance. In the treemix analysis, gene flow from *M. hancei* to *M. calvescens* was detected, and *M. matthewii* and *M. calvescens* were clustered as sister groups on the maximum likelihood tree. This same gene flow was confirmed by the Dsuit results, using the ABBA-BABA principle, finally confirming that *M. matthewii* is a hybridization between *M. hancei* and *M. calvescens*.

### 4.3 Bi-direction and asymmetrical hybridization

By employing phylogenetic analysis using chloroplast SNPs to determine the parentage of *M. matthewii*, we discovered evidence of bidirectional and asymmetrical hybridization. The chloroplast phylogenetic analysis showed that most hybrids clustered with *M. hancei*, while a few clustered with *M. calvescens*. This suggests that both *M. hancei* and *M. calvescens* could be maternal or potential paternal parents of *M. matthewii*. This different from the numerous fern hybridizations with defined parents proved in previous studies, such as *A. meishanianum*, a hybrid with *A. menglianense* as father and *A. malesianum* as mother (Zhang et al., 2014; Shang et al., 2016).Therefore, it is likely that *M. matthewii* is a product of bi-directional hybridization involving *M. hancei* and *M. calvescens*.

Furthermore, it was observed that despite the minimal contribution of plastid genetics, offspring resulting from the two types of hybridization still displayed morphological differences. For instance, *M. matthewii*, with *M. calvescens* as its mother, exhibited longer petioles (Luo, 2018). This bidirectional hybridization may have further enriched biodiversity. Similar to *M. matthewii*, samples of *M. krameri* are positioned on the branches of both *M. hancei* and *M. marginata* in the chloroplast phylogenetic tree (Luo et al., 2018). This positioning suggests a potential bi-directional hybridization between these two species. However, further verification is required through the collection of additional material.

Out of the 21 hybrid offspring detected, 17 had *M. hancei* as the mother, while only 4 had *M. calvescens*. This resulted in a ratio of 4.25:1, significantly deviating from the expected 1:1 ratio. This type of asymmetrical hybridization has been previously reported in hybridization studies of two North American *Dryopteris* hybrids. For example, a 7.1:1 ratio of c*arthusiana*: *intermedia* genome inheritance was found for D. × *triploidea*, and a 3.6:1 *cristata*: *intermedia* ratio was detected for D. × *boottii*. These deviations were driven by an array of reproductive traits, such as archegonial neck canals and spermatozoids (Testo et al., 2015).

## Data availability statement

The data presented in the study are deposited in the NCBI Sequence Read Archive (SRA, PRJNA1081188), accession numbers can be found in **Supplementary Table S1**.

## Author contributions

JL: Data curation, Formal Analysis, Investigation, Methodology, Writing – original draft, Writing – review & editing. HSha: Formal Analysis, Funding acquisition, Methodology, Project administration, Visualization, Writing – original draft, Writing – review & editing. ZX: Formal Analysis, Methodology, Software, Writing – review & editing. YW: Conceptualization, Resources, Writing – review & editing. XD: Resources, Supervision, Writing – review & editing. HShe: Project administration, Resources, Writing – review & editing. YY: Conceptualization, Funding acquisition, Project administration, Supervision, Writing – review & editing.

## Supplementary material

Supplementary Figure 1
**(A)** the variance contribution of each principal component in the PCA analysis; **(B)** the cross-validation error for each K in the admixture analysis.

Supplementary Table 1 The NCBI accession number, taxon identification (Latin name), specimen voucher and Location for the samples in this study.
