# Supplementary figures and images for: Genome-wide data reveal bi-direction and asymmetrical hybridization origin of a fern species Microlepia matthewii
Source: Front Plant Sci. 2024 Jul 8;15:1392990. doi: 10.3389/fpls.2024.1392990 (PMC11260791; doi:10.3389/fpls.2024.1392990)

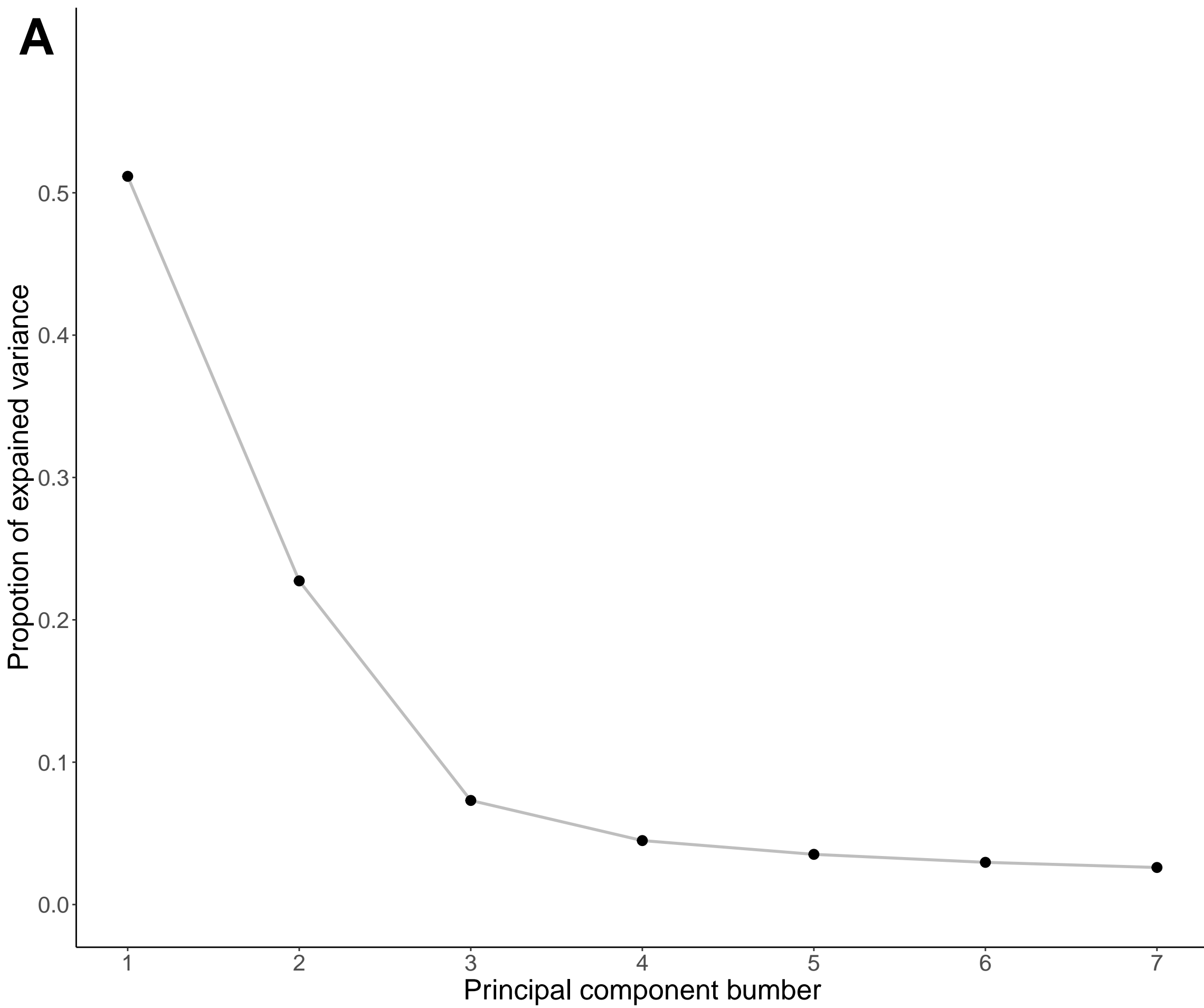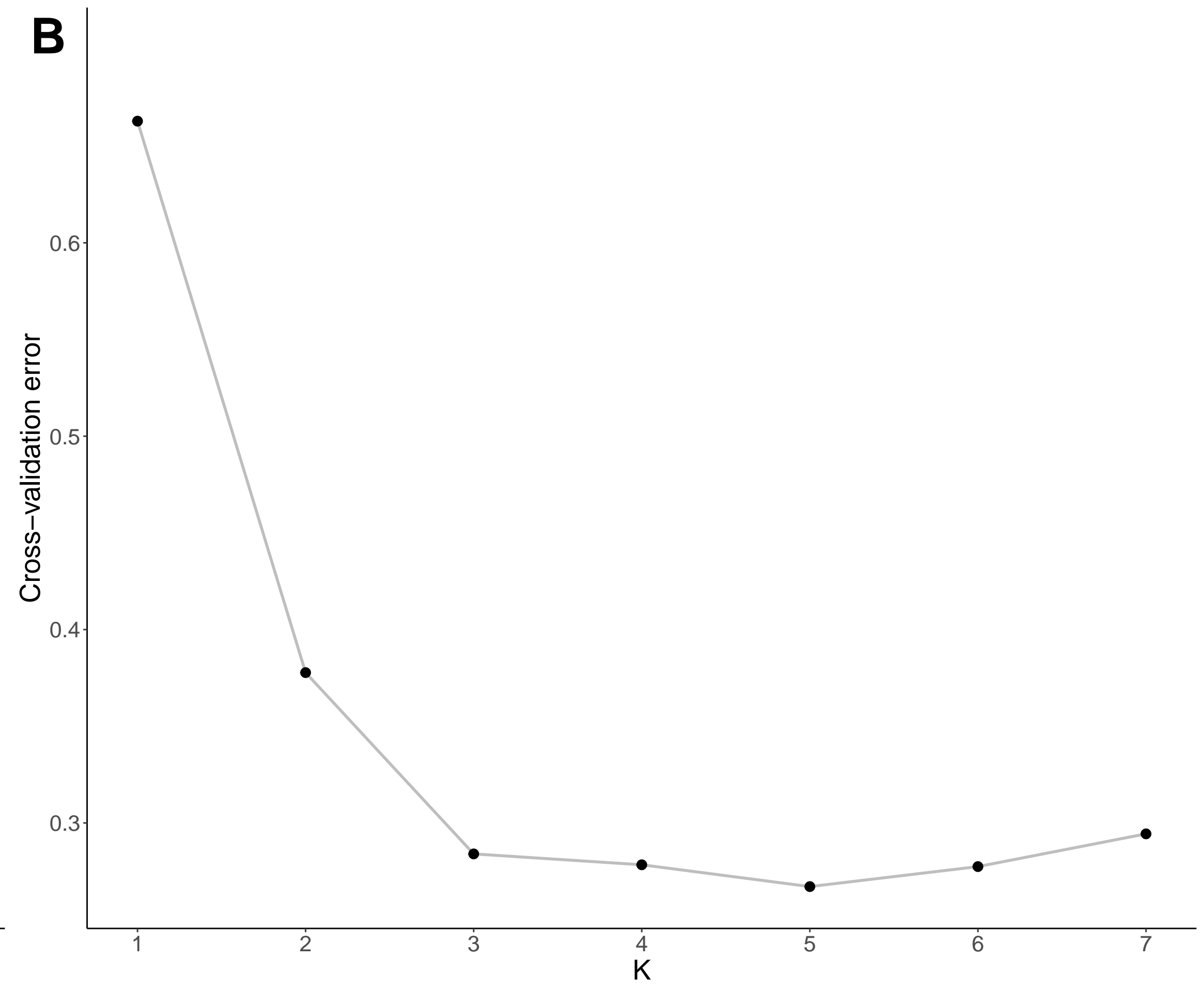

Supplement: Supplementary Figure 1 — (A) the variance contribution of each principal component in the PCA analysis; (B) the cross-validation error for each K in the admixture analysis. [file Image_1.pdf]
